# The prognostic value of six survival-related genes in bladder cancer

**DOI:** 10.1038/s41420-020-00295-x

**Published:** 2020-07-13

**Authors:** Shuting Cheng, Zhou Jiang, Jing Xiao, Huiling Guo, Zhengrong Wang, Yuhui Wang

**Affiliations:** Health Ministry Key Laboratory of Chronobiology, West China School of Basic Medical Sciences & Forensic Medicine, Sichuan University, Chengdu, P.R. China

**Keywords:** Tumour biomarkers, Prognostic markers

## Abstract

This study was conducted to identify genes that are differentially expressed in paracancerous tissue and to determine the potential predictive value of selected gene panel. Gene transcriptome data of bladder tissue was downloaded from UCSC Xena browser and NCBI GEO repository, including GTEx (the Genotype-Tissue Expression project) data, TCGA (The Cancer Genome Atlas) data, and GEO (Gene Expression Omnibus) data. Differentially Expressed Genes (DEGs) analysis was performed to identify tumor-DEGs candidate genes, using the intersection of tumor-paracancerous DEGs genes and paracancerous-normal DEGs genes. The survival-related genes were screened by Kaplan–Meier (KM) survival analysis and univariable Cox regression with the cutoff criteria of KM < 0.05 and cox *p*-value < 0.05. The risk model was developed using Lasso regression. The clinical data were analyzed by univariate and multivariate Cox regression analysis. Gene Ontology (GO) and KEGG enrichment analysis were performed in the DEGs genes between the high-risk and low-risk subgroups. We identified six survival-related genes, EMP1, TPM1, NRP2, FGFR1, CAVIN1, and LATS2, found in the DEG analyses of both, tumor-paracancerous and paracancerous-normal differentially expressed data sets. Then, the patients were classified into two clusters, which can be distinguished by specific clinical characteristics. A three-gene risk prediction model (EMP1, FGFR1, and CAVIN1) was constructed in patients within cluster 1. The model was applied to categorize cluster 1 patients into high-risk and low-risk subgroups. The prognostic risk score was considered as an independent prognostic factor. The six identified survival-related genes can be used in molecular characterization of a specific subtype of bladder cancer. This subtype had distinct clinical features of T (topography), N (lymph node), stage, grade, and survival status, compared to the other subtype of bladder cancer. Among the six identified survival-related genes, three-genes, EMP1, FGFR1, and CAVIN1, were identified as potential independent prognostic markers for the specific bladder cancer subtype with clinical features described.

## Introduction

Bladder cancer is a malignant tumor with high morbidity and high mortality. The steadily-rising incidence and prevalence make bladder cancer one of the most common urogenital cancers in the word. There were 549,393 new cases and 199,922 deaths reported worldwide in 2018^1^. The most common pathological type of bladder cancer is transitional cell carcinoma, with non-muscle-invasive bladder cancers (NMIBCs), and muscle-invasive bladder cancers (MIBCs). The treatment outcomes are very different for bladder cancer patients with MIBCs and NMIBCs^2^. MIBCs are usually associated with less favorable prognosis, lower 5-year survival rates, cancer progression, and metastasis. Patients with NMIBCs have much higher expected 5-year survival rate. However, NMIBCs could progress into MIBCs^3^. We conducted in silico study to improve the accuracy in prediction of 5-year patient survival rates for both types of bladder cancer.

Analysis of differentially expressed genes between conditions is an integral part of understanding the molecular basis of phenotypic variation in biology, including diseases^4^. Paracancerous tissue, the tissue adjacent to a solid tumor, is often used as a histologically normal, control sample in research studies on tumors. However, a recent study reported that the paracancerous tissue had quite distinct characteristics compared to either tumor tissue or histologically normal healthy tissue, suggesting a unique intermediate state between healthy tissue and tumor^5^. Additional essential information might be obscured when paracancerous tissue was used as a control instead of using healthy normal tissue.

In this study, we aimed to understand the differences in gene expression between tumor-adjacent and healthy tissue, and to determine potential prognostic value of the genes differentially expressed in paracancerous tissue. We first obtained the survival-related genes from RNA expression interaction of tumor-paracancerous DEGs genes and paracancerous-normal DEGs genes. Subsequently, we classified the bladder cancer patients with six survival-related genes by the consistent clustering algorithm and Lasso cox regression. The clusters and subgroups of patients were systematically analyzed with their clinicopathological features, and the difference between subgroups was analyzed by GO and KEGG enrichment.

## Results

### Dataset included in the analyses

For gene transcriptome data, we analyzed a total of 411 cases of TCGA bladder cancer, 19 cases of TCGA paracancerous tissue, nine cases of GTEx healthy bladder, 36 cases of GEO bladder cancer, 29 cases of GEO paracancerous tissue, and five cases of GEO healthy bladder. There were only 345 cases of available TCGA clinical information. Among the clinical information, the metastasis could not be measured in more than half of the cases (176 cases). No available clinical information on GEO data was found.

### Identification of DEGs genes in bladder cancers

Using Limma and edgeR packages, 1413 tumor-paracancerous DEGs RNA sequences and 3223 paracancerous-normal DEGs RNAs were identified in the TCGA-GTEx dataset, and 771 tumor-paracancerous DEGs RNAs and 2963 paracancerous-normal DEGs RNAs in the GEO dataset. After overlapping all data, a total of 60 genes were selected as potential tumor-DEGs genes.

### The survival-related genes

**Fig. 1:**
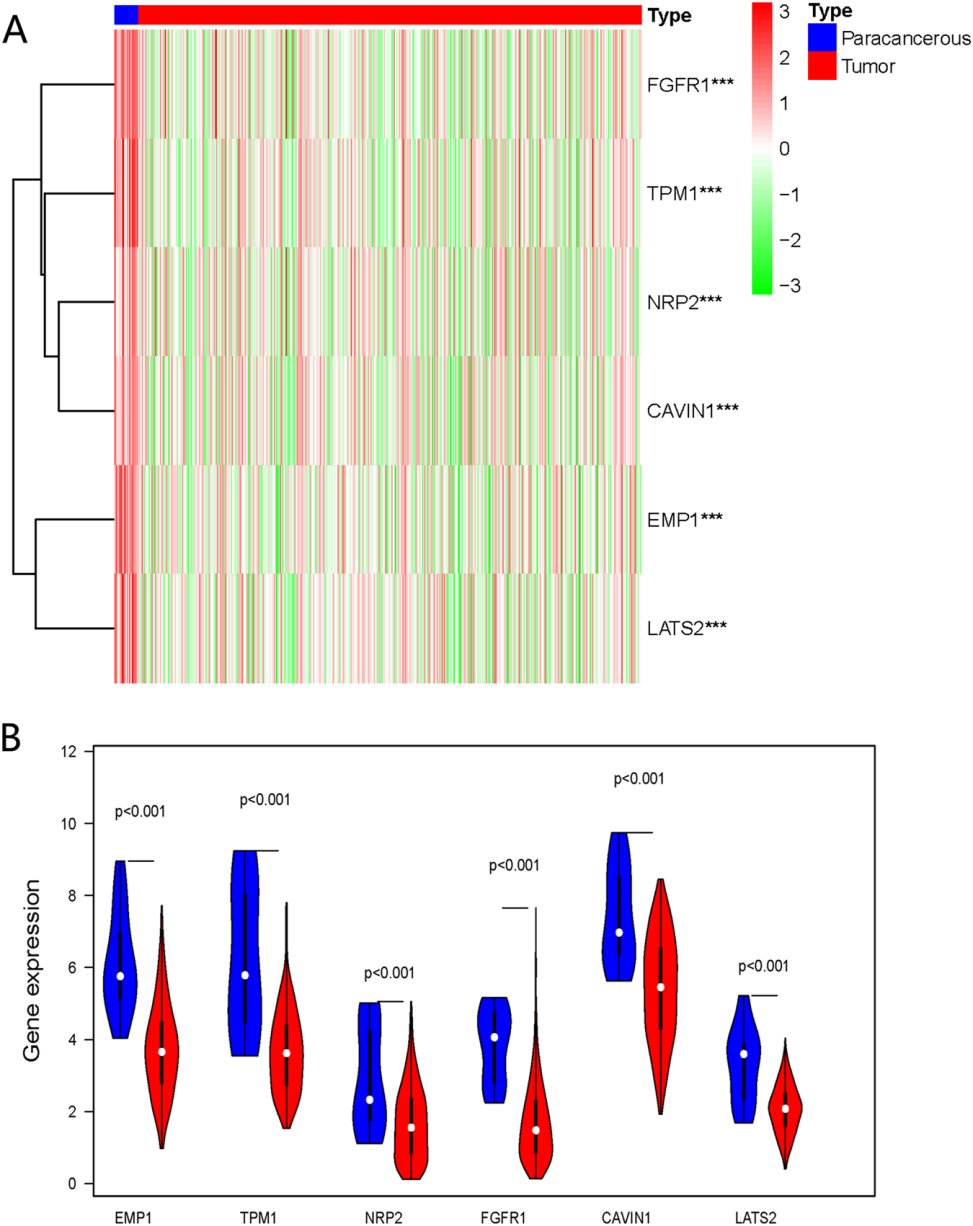
Expression of survival-related genes in bladder cancer. **a** Heatmap of the expression of six survival-related genes in paracancerous and tumor tissue of bladder cancer. Red color represents a high expression of the gene, while the green color represents low expression. ***p < 0.001. **b** The violin diagram showed the differential expression of six survival-related genes in bladder cancer and paracancerous tissue. Blue color represents paracancerous tissue, and the red color represents the bladder tumor.

The expression of six survival-related genes in the TCGA database was shown in Fig. 1, i.e., EMP1 (epithelial membrane protein 1), TPM1 (tropomyosin 1), NRP2 (neuropilin 2), FGFR1 (fibroblast growth factor receptor 1), CAVIN1 (caveolae associated protein 1), and LATS2 (large tumor suppressor kinase 2). The expression of the six genes in tumor tissues was downregulated significantly compared to the paracancerous tissue (*p* < 0.001).

### The characteristics of two clusters

**Fig. 2:**
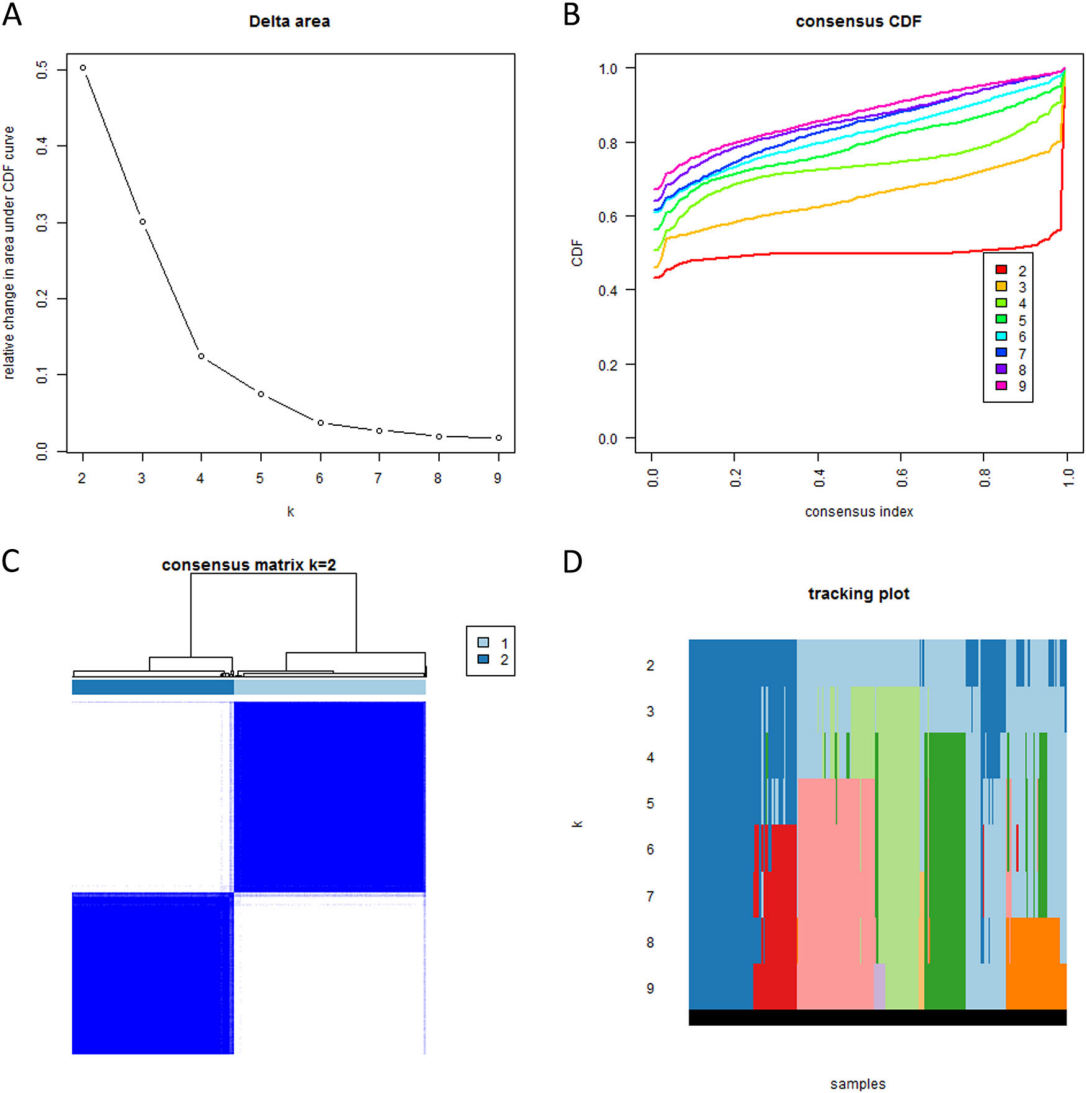
Consistent cluster analysis of bladder cancer. **a** The relative change in area under the cumulative distribution function (CDF) curve from 2 to 9 of *k*. **b** The consensus CDF plot corresponding to the consensus matrices when *k* is between 2 and 9. **c** The correlation between groups when *k* is equal to 2. **d** The distribution of samples when *k* is between 2 and 9.

**Fig. 3:**
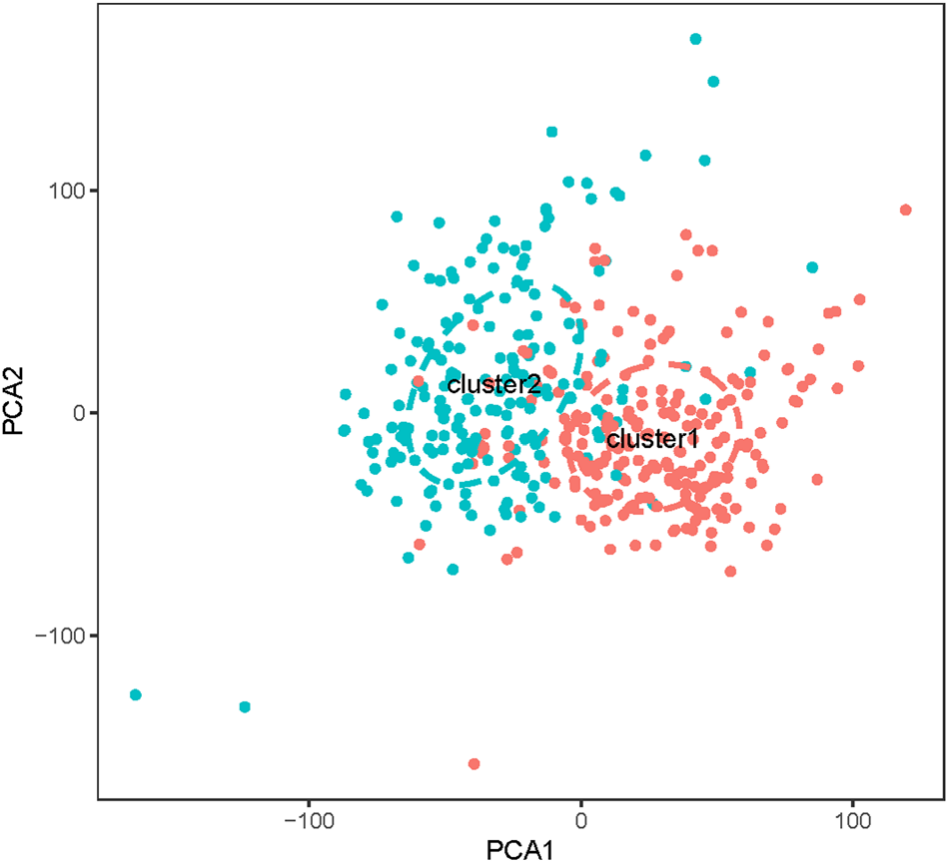
PCA of 2 clusters on the total gene expressions. PCA was performed in TCGA RNA-Seq FPKM data of bladder cancer. The scattered dots showed the distribution of genes in the 2 cluster groups. Red dots represented cluster 1 group, while green dots represented cluster 2 group.

Based on the expression of the survival-related genes in TCGA, patients were clustered into two groups by consistent clustering (Fig. 2). The principal component analysis (PCA) showed that RNA expression in two clusters was mainly specific (Fig. 3).

**Fig. 4:**
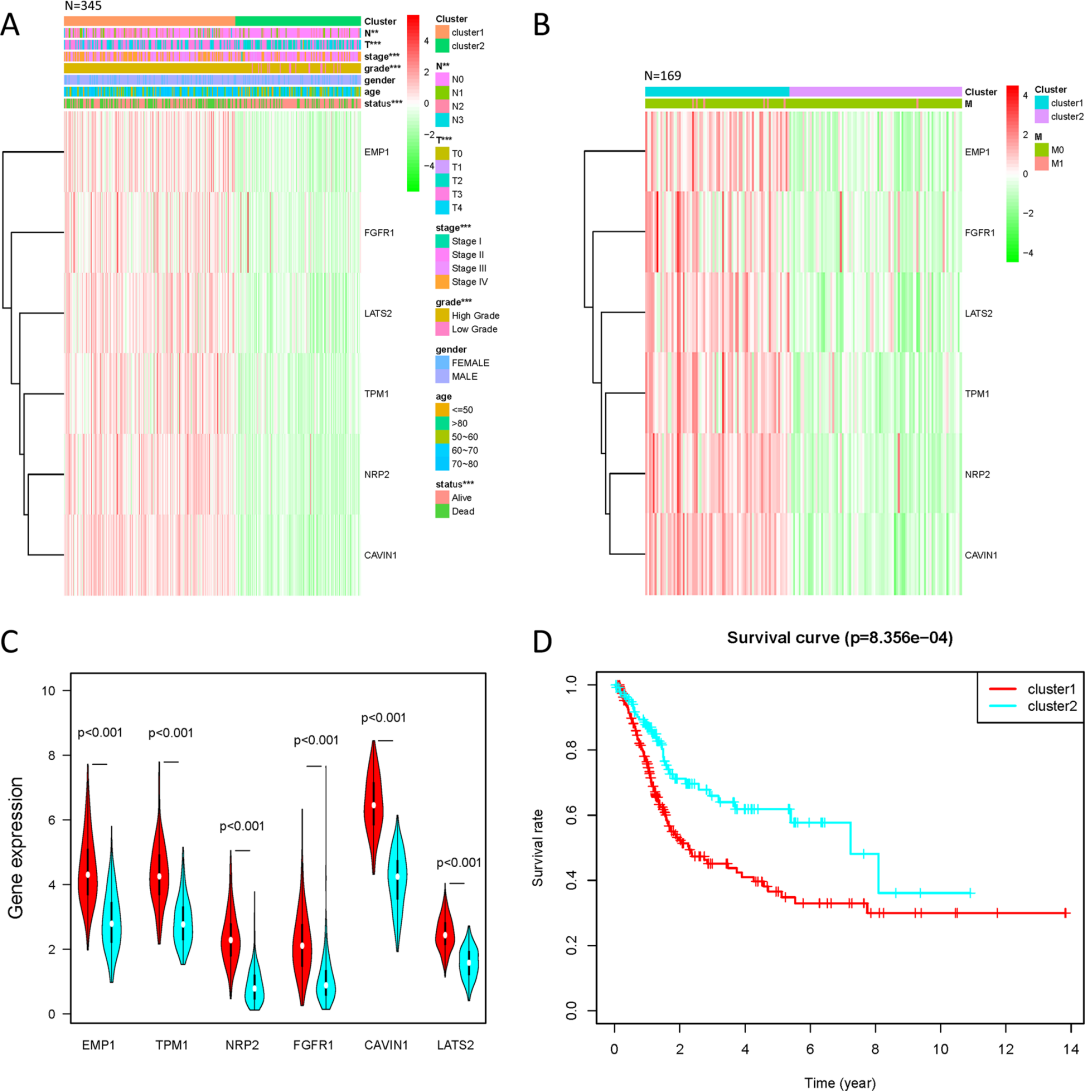
Comparison of clinical characteristics and expression of survival-related genes between 2 cluster groups of patients divided by consistent cluster analysis. **a** The clinicopathological features, which included N, T, stage, grade, gender, age, and survival status, and the expressions of survival-related genes distributed in the heatmap of 2 defined clusters of 345 patients. **p < 0.01, ***p < 0.001. **b** The clinicopathological features of M and the heatmap of survival-related genes distributed in the two defined clusters of 169 patients. **c** The violin diagram shows the comparison of the median values of six survival-related genes in 2 clusters of bladder cancer, and the location of the white dots represented the median value of expressions. **d** Comparison of overall survival rates between 2 cluster groups of bladder cancer.

The clinical information of gender, age, N, T, stage, grade, and survival status were analyzed in 345 patient cases; all of them had a significant difference between two clusters except gender and age (Fig. 4a). Prognostic factor M, which was analyzed in 169 cases of patients with complete clinical information, had no significant difference between two clusters (Fig. 4b). By comparing the expression of survival-related genes in cluster 1 and 2 (Fig. 4a–c), the difference in expression of all six survival-related genes were significantly higher in cluster 1 (*p* < 0.001). The overall survival rate of cluster 2 was significantly higher than cluster 1 (*p* = 8.356 × 10^−4^, Fig. 4d).

### Predictive value of the three-survival-related-gene risk model in prognosis

**Fig. 5:**
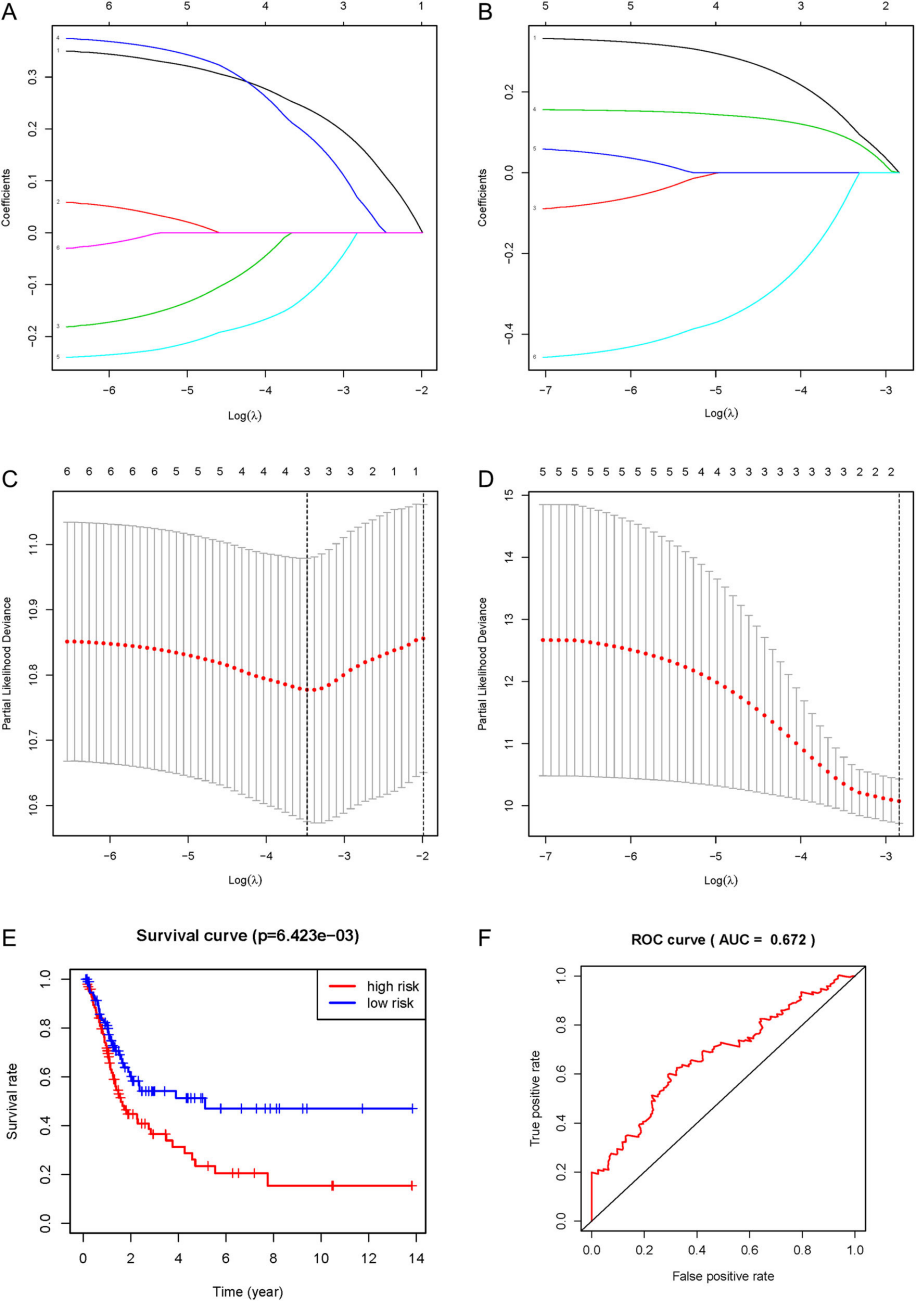
Construction of the risk model in 2 clusters and its survival verification. The risk score model was constructed using the LASSO regression analysis along with 10-fold cross-validation. **a** Processes of LASSO Cox model fitting in cluster 1 group. **b** Processes of LASSO Cox model fitting in cluster 2 group. The LASSO coefficient of each gene associated with the overall survival was represented as a curve. **c** The partial likelihood deviance with changing of log (*λ*) in cluster 1 was plotted. **d** The partial likelihood deviance with changing of log (*λ*) in cluster 2 was plotted. The number corresponded to the point with the smallest cross-verification error was the gene numbers included in the Lasso regression risk model. **e** The overall survival rate curves of high-risk subgroup and low-risk subgroup. **f** The ROC curve showed the accuracy of the predictive survival model by the area under the curve (AUC).

With six survival-related genes for analysis, a risk model was constructed in cluster 1 to divide patients into high-risk and low-risk subgroups (Fig. 5a, c). The result showed that three survival-related genes, EMP1, FGFR1, and CAVIN1, were included in the risk model, with risk coefficients were 0.240, 0.191, and −0.121, respectively. The survival rates of the high-risk and low-risk subgroups were compared, finding a significant difference in overall survival between the two subgroups (*p* = 6.423 × 10^−3^, Fig. 5e). The accuracy of the predictive survival model was confirmed by the area under the curve (AUC = 0.672), as shown in Fig. 5f.

**Fig. 6:**
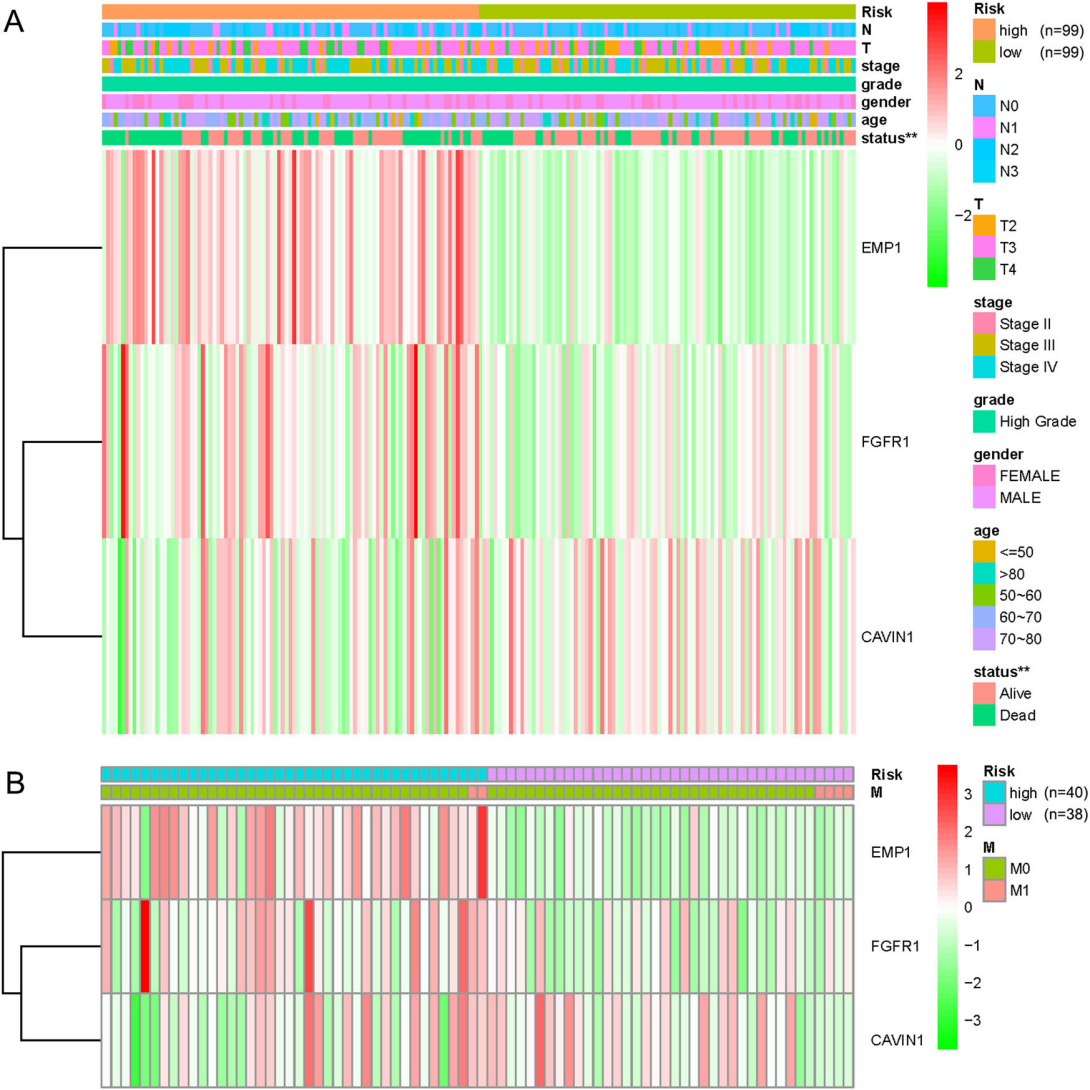
Comparison of clinicopathological characteristics and expression of EMP1, FGFR1, and CAVIN1 between the high-risk and low-risk subgroups. **a** The clinicopathological features, which included N, T, stage, grade, gender, age, and survival status, and the expressions of EMP1, FGFR1, and CAVIN1 distributed in the heatmap of 2 risk subgroups of 198 patients. **p < 0.01. **b** The clinicopathological features of M and the expressions of EMP1, FGFR1, and CAVIN1 distributed in the heatmap of 2 risk subgroups of 78 patients.

The clinical characteristics and the expression of EMP1, FGFR1, and CAVIN1 were also compared between the two subgroups (Fig. 6). The subgroups cannot be distinguished by the clinical characteristics. However, both EMP1 and FGFR1 expression in the high-risk group were significantly higher than those in the low-risk group (*p* < 0.001), while CAVIN1 was less expressed in the high-risk group (*p* < 0.05).

**Fig. 7:**
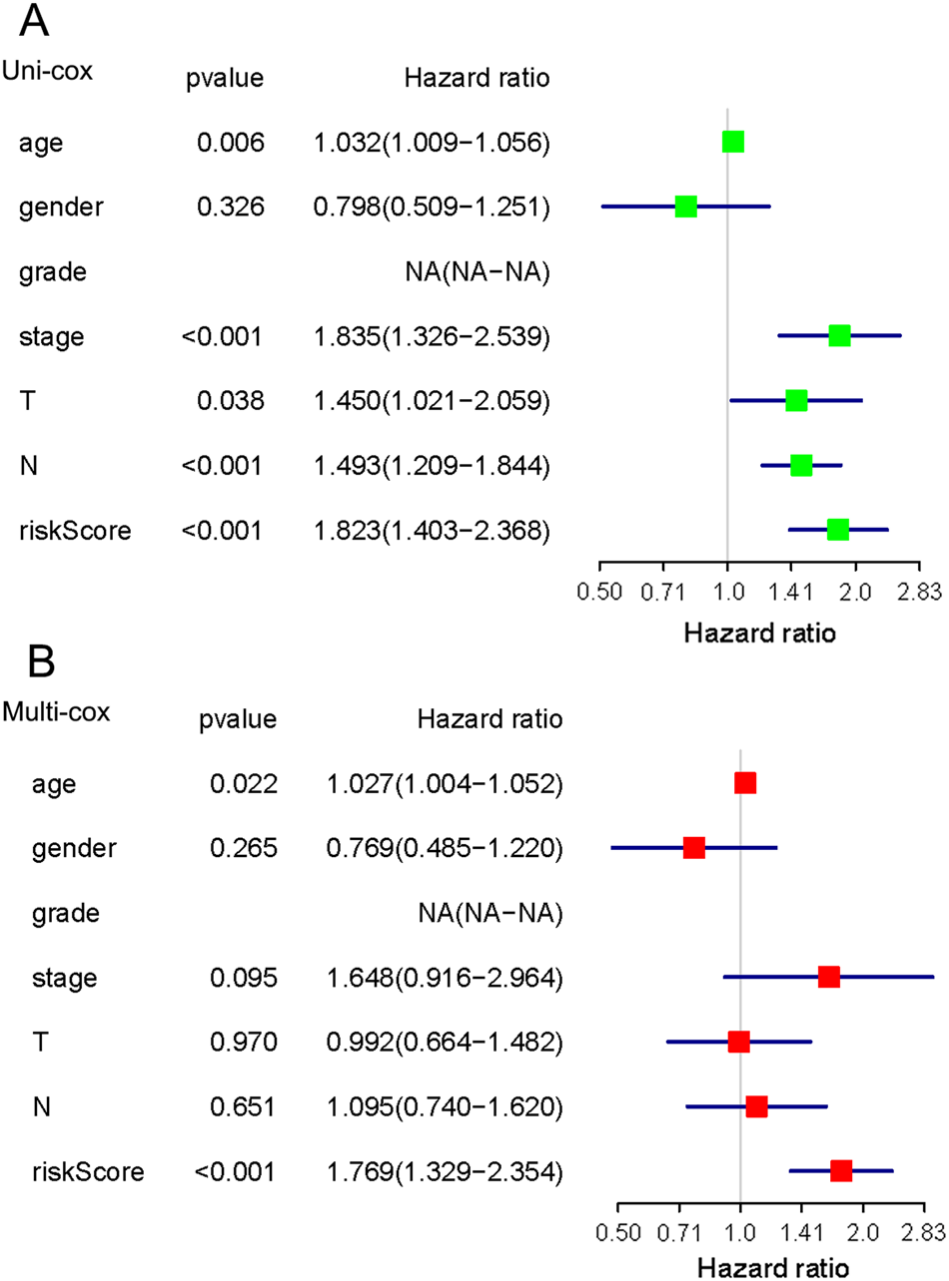
The independent prognostic analysis on risk score and clinicopathological features. **a** The hazard ratio (HR) and 95% confidence interval of risk score and all clinical features were calculated by univariate regression analysis. **b** The hazard ratio (HR) and 95% confidence interval of risk score and all clinical features were calculated by multivariate regression analysis. The factor in the analysis could be considered as an independent prognostic factor when both *p* values were <0.05 in **a**, **b**.

The Hazard ratio (HR) of different clinical features (with the exception of M) in the two subgroups was further analyzed. HR of grade and its 95% confidence interval could not be obtained because there was only high grade in this cluster. As shown in Fig. 7a, b, the resulting prognostic factors were estimated by univariate and multivariate Cox regression analysis. It showed that the *p* values of risk score were all <0.001, and the HR values were >1.

### GO and KEGG pathways enriched in high-risk and low-risk DEGs genes in the cluster 1

**Fig. 8:**
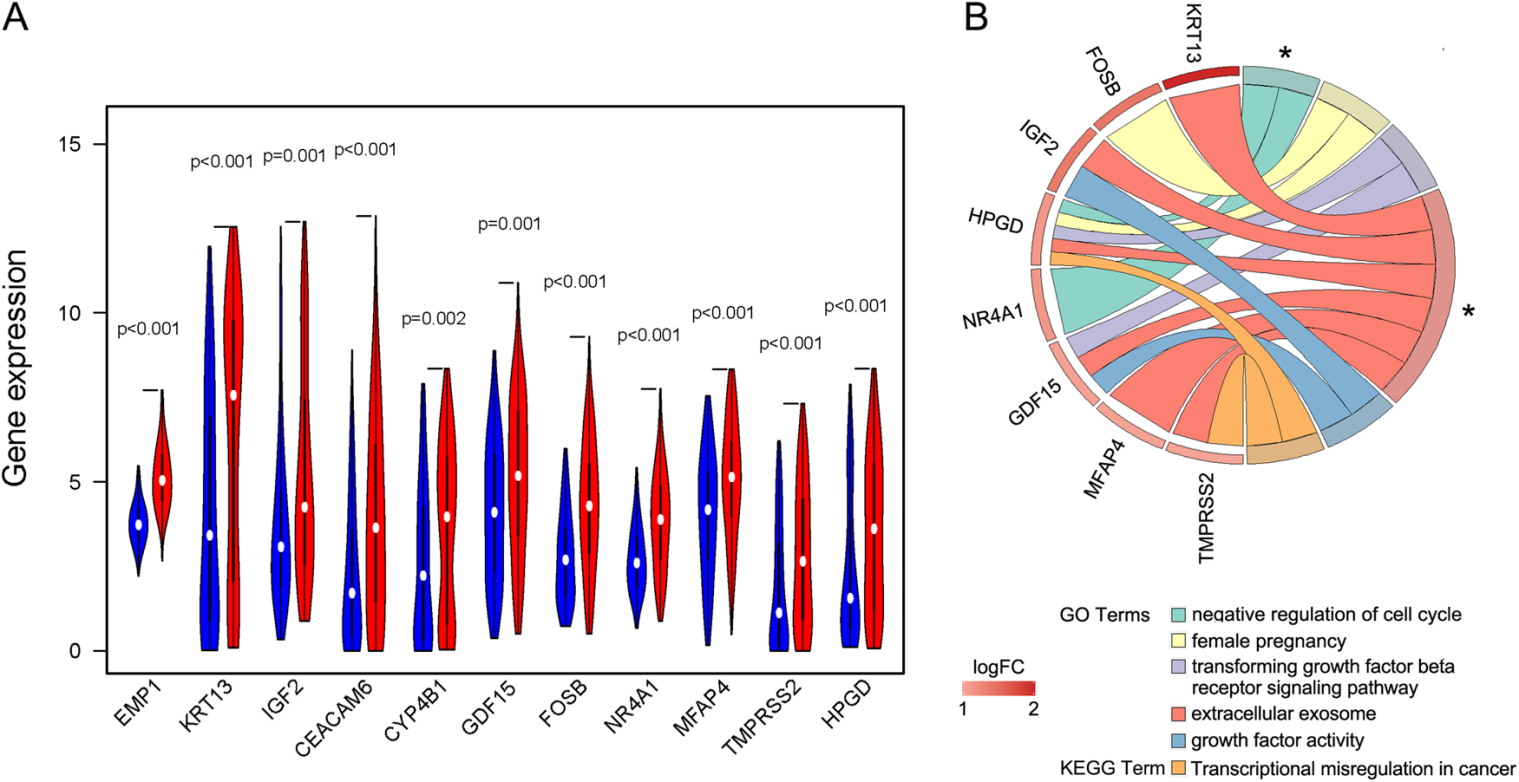
The enrichments of DEGs genes obtained between the high-risk and low-risk subgroups of cluster 1. **a** The violin diagram showed the differential expression of 11 DEGs genes from high-risk and low-risk subgroups of cluster 1. Blue color represents the low-risk subgroup, and the red color represents the high-risk subgroup. **b** The Chord diagram showed the GO and KEGG pathway terms which were enriched in high-risk and low-risk DEGs genes of cluster 1. The asterisks marked the terms with a *p-*value < 0.05.

Eleven DEGs genes were selected from the differential gene analysis between the high-risk and low-risk subgroups of the cluster 1 (Fig. 8a). After the functional annotation of the DEGs genes, a list of GO and KEGG pathway terms was enriched (Fig. 8b). However, only two GO terms, i.e., “GO:0045786~negative regulation of cell cycle” and “GO:0070062~extracellular exosome”, were estimated to be with a *p*-value < 0.05.

## Discussion

Bladder cancer is one of the most common urogenital cancers in the word. It carries a high risk of morbidity and mortality and is one of the main causes of death. It is acknowledged that NMIBCs has a higher survival rate than MIBCs, and the survival rate increases when it progresses to MIBCs^3^. Most research focused on bladder cancer has been carried out using paracancerous tissue as normal control. However, it has been suggested that some additional information might be obscured when using paracancerous tissue as a control^5^.

The goal of our study was to reveal the potential value of paracancerous tissue in predicting the prognosis of bladder cancer patients. Sixty DEGs were identified in paracancerous tissue comparing to both, tumor tissue and normal control. Since the GEO data had no clinical information, the survival analysis and clinical correlation analysis were conducted in TCGA data. Lastly, we identified six survival-related genes (EMP1, TPM1, NRP2, FGFR1, CAVIN1, and LATS2) and used this gene-set to group the bladder cancer patients into two clusters, while three survival-related genes (EMP1, FGFR1, and CAVIN1) were used to estimate the prognostic risk for the patients grouped in cluster 1.

All six selected survival-related genes (EMP1, TPM1, NRP2, FGFR1, CAVIN1, and LATS2) were reported to play a positive role in disseminating cancer cells or invasion in many kinds of cancers^6–11^. EMP1 belongs to the peripheral myelin protein 22-kDa (PMP22) gene family. It promotes glioblastoma progression through the PI3K/AKT/mTOR signaling pathway^6^, and the unusual expression of EMP1 has been revealed in most tumor types^12^. TPM1 gene is a member of a tumor-associated protein family (the tropomyosin family) - it plays an important role in tumor-specific variations of actin filament aggregation via stress fiber modulation and actin cytoskeleton modification^7^. LATS2 gene belongs to the LATS tumor suppressor family. It encodes a serine/threonine protein kinase, reported to be involved in Hippo signaling pathway^11^. Similarly, the essential caveolar gene CAVIN1 is also involved in the Hippo pathway^10^. It appears the Hippo signaling pathway might be associated with the particular cluster 1 of bladder cancer. However, further experimental verification is needed.

Moreover, NRP2 and FGFR1 expressions were explicitly associated with cancer survival in bladder cancer^13,14^. NRP2 is a member of the membrane-associated neuropilin family and has been reported to be expressed during macrophage differentiation and facilitated tumor growth^8^. The NRP2 gene is reported as a prognostic indicator for reduced survival in bladder cancer patients^15^. FGFR1 is a member of the fibroblast growth factor receptor (FGFR) family, and is known to be involved in the progression of different cancer types, including bladder cancer^9^.

In our analysis, all six survival-related genes were expressed at significantly lower levels in tumor tissue (*p* < 0.001, Fig. 1). The PCA showed that RNA expression in these two groups were mainly specific (Fig. 3). All six survival-related genes were significantly higher expressed in cluster 1 (*p* < 0.001, Fig. 4a–c). Also, all the clinicopathological features (with the exception of M, gender, and age) were significantly different between the two clusters (Fig. 4a, b). It indicates that this kind of clustering can distinguish bladder cancer in the aspects of N, T, stage, grade, and survival status. Based on the survival curve of the two clusters, we concluded that patients in cluster 1 had lower survival rates (Fig. 4d). The six survival-related genes, which were the basis for clustering, played a negative role in the survival of patients. This is in accordance with the previously reported studies of these genes^6–11^.

We intended to construct the risk models including survival-related genes for each of the two clusters, however, we successfully outlined the risk model only for the cluster 1, in which EMP1, FGFR1, and CAVIN1 were included (Fig. 5a, c). It was not feasible to construct the risk model for cluster 2 because we could not define the model by probing any of the six survival-related genes (Fig. 5b, d). In the risk model for cluster 1, the low-risk subgroup had significantly better survival than the high-risk subgroup (Fig. 5e), and the prediction efficiency of the curve was good (Fig. 5f). The expressions of EMP1, FGFR1, and CAVIN1 were related to the risk model; however, this risk model did not corelate with the clinical features (Fig. 6). Significantly higher expressions of EMP1 and FGFR1 (*p* < 0.001) and relative lower expression of CAVIN1 (*p* < 0.05) in the high-risk subgroup suggested that the risk was associated with altered expression of these three genes. The results of univariate and multivariate Cox regression analysis indicated that the risk score obtained by our model can be used as an independent prognostic factor for overall survival in a specific cluster of bladder cancer patients (Fig. 7). This independent prognostic factor was not related to the clinicopathological features of stage, grade, gender, age, survival status, T, N, or M.

To explore the difference between the low-risk subgroup and the high-risk subgroup, we identified their DEGs and uploaded the DEGs to the DAVID tool for a GO and KEGG pathway enrichment analysis. It appears that the risk score was related to the negative regulation of cell cycle and the component of extracellular exosome. NR4A1 (nuclear receptor subfamily 4 group A member 1) and HPGD (15-hydroxyprostaglandin dehydrogenase) were involved in the negative regulation of cell cycle. While the component of extracellular exosome were associated to the expression levels of TMPRSS2 (transmembrane serine protease 2), KRT13 (keratin 13), IGF2 (insulin-like growth factor 2), MFAP4 (microfibril associated protein 4), GDF15 (growth differentiation factor 15), and HPGD.

Among these enriched genes, IGF2 is reported to be frequently overexpressed in bladder cancer^16^. Inhibition of the gene expression of IGF2 and IGF2-mediated PI3K/AKT/mTOR signaling pathway could suppress the development of bladder cancer^17^. DNA methylation of GDF15 was significantly high in bladder cancer. Therefore, this gene could be used as an epigenetic biomarker to identify bladder cancer^18^. DNA demethylation of GDF15 would upregulate its expression, which led to a suppression of cell proliferation, invasion, and tumorigenesis in bladder carcinoma cells^19^. It was also reported that methylation of KRT13 had a close relationship with high-grade non-invasive bladder cancer^20^. That is to say, lower expressions of IGF2, GDF15, and KRT13 was associated with lower cell proliferation, invasion, and tumorigenesis of bladder cancer. These findings were consistent with our results, in which IGF2, GDF15, and KRT13 expression were significantly decreased in the low-risk subgroup. Interestingly, it was reported that an increase of HPGD would suppress cell proliferation and invasion in bladder cancer^21^, which implied that patients with a high expression of HPGD might have a better survival rate. However, in our analysis, an increased expression of HPGD was found in the high-risk subgroup, with a worse overall survival rate.

The current study has identified promising models for characterization of a specific subtype of bladder cancer and independent estimation of the prognostic risk in this particular subtype. However, the generalizability of these results is subject to evident limitations. One limitation of this study is that our sample size for normal control was too small, some potential association could not be revealed. Another source of limitation is that we did not have experimental verification for the findings in this study. Therefore, further research with larger sample sizes of normal control and more experiments is warranted.

While there are limitations in our study, there are also undoubted strengths. We do believe that this set of six identified survival-related genes could be a useful tool for identification of a particular subtype of bladder cancer. This subtype of bladder cancer had quite different clinical features of T, N, stage, grade, and survival status from the other subtype of bladder cancer. The survival rate of patients was negative related to the expressions of the six survival-related genes. In this particular subtype, the prognosis of bladder cancer can be independently evaluated by a risk model composed of EMP1, FGFR1, and CAVIN1 genes. The high-risk and low-risk subgroups, which were classified by the 3-gene prognostic factor model, might have a different biological process on the negative regulation of cell cycle and a different cellular component in extracellular exosome. These might be the reason account for their different prognostic factors.

## Materials and methods

### Data preparation

The Fragments per Kilobase of transcript per Million mapped reads (FPKM) for the bladder cancer RNA-Seq data from TCGA and GTEx were downloaded from the UCSC Xena browser platform^22^. The latest clinical data of TCGA was downloaded from GDC (Genomic Data Commons), and patients with unavailable clinical information were excluded. After combining the data from GTEx and TCGA, genes were selected by FPKM > 0 for further analysis.

GEO RNA-Seq raw data of bladder cancer (GSE133624) and normal healthy bladder in GSE120795 were downloaded from the NCBI GEO SRA database. After quality control for the sequence data, raw counts were obtained by alignment to “Homo_sapiens.GRCh38.96.gtf” by the read-count program featureCounts^23^. Datasets were merged according to the Ensembl IDs. The filtering criteria for the merged dataset were raw counts > 0 and CPM > 1. Next, the gene annotation was performed with “hsapiens_gene_ensembl” dataset by the biomaRt R package^24,25^.

### Analysis of differentially expressed genes

The Limma (Linear Models for Microarray Data) R package^26^ and edgeR (Empirical Analysis of Digital Gene Expression Data in R) R package^27,28^ were used to identify the tumor-paracancerous DEGs genes and paracancerous-normal DEGs genes respectively in the FPKM and count data. In the screening, the FDR value < 0.05 and |logFC | > 1 cutoff criterion was applied. Tumor-DEGs genes were obtained by overlapping the tumor-paracancerous DEGs genes and paracancerous-normal DEGs genes from TCGA-GTEx and GEO datasets. The high-low risk DEGs genes (DEGs genes between high-risk subgroup and low-risk subgroup) were also identified by the Limma package, using FDR value < 0.05, and |logFC | > 1 cutoff criteria.

### Kaplan–Meier survival analysis and univariable Cox regression

Patient’s survival time and status, combined with tumor-DEGs genes, were used for the KM survival analysis and univariable Cox regression, performed with survival R package^29,30^. The cutoff criterion was set to KM < 0.05 and cox *p*-value < 0.05 for screening of the survival-related gene at overall survival.

### Clustering of patients

ConsensusClusterPlus R package^31^ was executed to evaluate the stability of clustering by the consistent clustering algorithm. Small clusters were excluded in the clustering data. The final clustering number of samples was determined according to the slow growth rate of cumulative distribution function (CDF) value and high correlation within the group.

### Principal component analysis

PCA for all Tumor-DEGs genes was performed by Limma R package^26^. The ggplot2 R package^32^ was used to visualize the results.

### Lasso regression

For the six survival-related genes, the Lasso regression algorithm was executed for each of the two patient clusters to develop a potential risk model and to evaluate the risk characteristics by using the glmnet R package^33^. The risk genes were identified among the survival-related genes through the minimum standard, and their coefficients were calculated. The best penalty parameter *λ*, related to the minimum ten times cross-verification in the training set, was selected to obtain the risk score. Consistent with the median risk score, patients were further divided into high-risk and low-risk subgroups. Using the survivalROC R package^34^, the area under the Receiver Operating Characteristic (ROC) curve was calculated to detect the accuracy of the predictive survival model^35^.

### Analysis of the risk characteristics and prognosis in the cluster 1

The survival analysis was performed and the survival curves in different groups were compared by the log-rank test. The prognostic factors (i.e., stage, grade, gender, age, survival status, T, N, and M) and the risk genes in those groups/subgroups were investigated and compared by the *χ*^2^ test. The potential independent prognostic factor was explored in stage, gender, age, TNM (topography, lymph node, and metastases) classification, and risk score by univariate and multivariate Cox regression analysis. These programs were executed using the survival R package^29,30^.

### Enrichment analysis

Biological function of high-/low-risk DEGs genes of cluster 1 was evaluated with GO and KEGG enrichment analyses, performed using DAVID Functional Annotation Bioinformatics Microarray Analysis method (http://david.abcc.ncifcrf.gov/home.jsp)^36,37^.

### Statistical analysis

The expression of survival-related genes in tumor tissues and paracancerous tissues was compared by one-way ANOVA. The clinical characteristics and survival-related genes of different groups were compared by the *χ*^2^ test. Kaplan–Meier method was used for the bilateral logarithmic rank test in overall survival analysis. All tests were two-sided, and *p* values < 0.05 were considered statistically significant. All the statistical analyses in this study are implemented by R v3.6.1 (https://www.r-project.org/).
